# Biomarkers of inflammation and innate immunity in atrophic nonunion fracture

**DOI:** 10.1186/s12967-016-1019-1

**Published:** 2016-09-06

**Authors:** Dominique de Seny, Gaël Cobraiville, Pierre Leprince, Marianne Fillet, Charlotte Collin, Myrielle Mathieu, Jean-Philippe Hauzeur, Valérie Gangji, Michel G. Malaise

**Affiliations:** 1 Laboratory of Rheumatology, Department of Rheumatology, GIGA Research, University of Liège, Tour GIGA, +2, CHU, 4000 Liège, Belgium; 2 GIGA-Neurosciences, University of Liège, 4000 Liège, Belgium; 3 Laboratory for the Analysis of Medicines, Department of Pharmacy, CIRM, University of Liège, 4000 Liège, Belgium; 4 Laboratory of Bone and Metabolic Biochemistry, Department of Rheumatology, Université Libre de Bruxelles (ULB), 1000 Brussels, Belgium; 5 Department of Rheumatology and Physical Medicine, Hôpital Erasme, Université Libre de Bruxelles (ULB), 1000 Brussels, Belgium

**Keywords:** Proteomics, Biomarkers, 2D-DIGE, SELDI, Nonunion, Innate immunity, Hepcidin, Complement, Apolipoprotein, S100A8

## Abstract

**Background:**

Nonunion is a failure of healing following a bone fracture. Its physiopathology remains partially unclear and the discovery of new mediators could promote the understanding of bone healing.

**Methods:**

Thirty-three atrophic nonunion (NU) patients that failed to demonstrate any radiographic improvement for 6 consecutive months were recruited for providing serum samples. Thirty-five healthy volunteers (HV) served as the control group. Proteomics studies were performed using SELDI-TOF–MS and 2D-DIGE approaches, associated or not with Proteominer® preprocessing, to highlight biomarkers specific to atrophic nonunion pathology. Peak intensities were analyzed by two statistical approaches, a nonparametric Mann–Whitney U tests (univariate approach) and a machine-learning algorithm called extra-trees (multivariate approach). Validation of highlighted biomarkers was performed by alternative approaches such as microfluidic LC–MS/MS, nephelometry, western blotting or ELISA assays.

**Results:**

From the 35 HV and 33 NU crude serum samples and Proteominer® eluates, 136 spectra were collected by SELDI-TOF–MS using CM10 and IMAC-Cu^2+^ ProteinChip arrays, and 665 peaks were integrated for extra-trees multivariate analysis. Accordingly, seven biomarkers and several variants were identified as potential NU biomarkers. Their levels of expression were found to be down- or up-regulated in serum of HV vs NU. These biomarkers are inter-α-trypsin inhibitor H4, hepcidin, S100A8, S100A9, glycated hemoglobin β subunit, PACAP related peptide, complement C3 α-chain. 2D-DIGE experiment allowed to detect 14 biomarkers as being down- or up-regulated in serum of HV vs NU including a cleaved fragment of apolipoprotein A-IV, apolipoprotein E, complement C3 and C6. Several biomarkers such as hepcidin, complement C6, S100A9, apolipoprotein E, complement C3 and C4 were confirmed by an alternative approach as being up-regulated in serum of NU patients compared to HV controls.

**Conclusion:**

Two proteomics approaches were used to identify new biomarkers up- or down-regulated in the nonunion pathology, which are involved in bone turn-over, inflammation, innate immunity, glycation and lipid metabolisms. High expression of hepcidin or S100A8/S100A9 by myeloid cells and the presence of advanced glycation end products and complement factors could be the result of a longstanding inflammatory process. Blocking macrophage activation and/or TLR4 receptor could accelerate healing of fractured bone in at-risk patients.

**Electronic supplementary material:**

The online version of this article (doi:10.1186/s12967-016-1019-1) contains supplementary material, which is available to authorized users.

## Background

Five to ten percent of fractures do not consolidate spontaneously after plaster cast immobilization or surgical intervention. Unconsolidated fractures are defined as nonunion fractures that have not made any progression toward healing 6 months after the fracture-related injury. Nonunion fracture represents a major socioeconomic issue mainly affecting young population and being particularly debilitating.

Two main forms of radiographic nonunion exist: atrophic nonunion for which there is no evidence of callus formation after 6 months of fracture and hypertrophic nonunion for which there is abnormal callus formation. Atrophic nonunion result from a failure of normal cellular response necessary for bone reconstruction [1]. Several studies have demonstrated that the pool of bone marrow-derived mesenchymal stem cells (hMSCs) in nonunions was decreased and their proliferation delayed [1–3]. However, once committed, hMSCs from nonunions were able to proliferate, differentiate into osteoblastic cells and mineralize in vitro as efficiently as hMSCs from healthy subjects [2]. Hypertrophic nonunion would rather appear following instability at the site of fracture. Poorly vascularized bones as scaphoid hand, talus, tibia, ulna or hip seem to be more often the seat of nonunion. Angiogenesis is necessary to promote revascularization of the injured site. New vessels enhance mobilization of osteoprogenitor cells and of growth factors involved in osteogenesis.

Risk factors for nonunion can be divided as patient-dependent factors (tobacco use, alcohol abuse, NSAID, malnutrition, diabetes, vascular disease, previous radiation therapy, hypothyroidism and vitamin D deficiency) or patient–independent factors (comminution, poor cortical apposition, interposed soft tissue, soft tissue damage, bone loss, quality of surgical treatment and infection) [4]. However, biological mediators can also be causative factors of nonunion. Several bone turnover markers such as bone-specific alkaline phosphatase [5–8], osteocalcin [5, 6, 9] and fragments of type-I and type-III procollagen [7] have been suggested as potential markers of nonunion. Levels of leptin, interleukin-6, platelet-derived growth factor-BB, stem cell factor and insulin-like growth factor-1 were found altered in nonunion compared to healthy volunteers [2]. Osteoprotegerin serum level was increased in atrophic nonunion shaft fractures [10].

Today there is a real need for a better understanding of the physiology and pathophysiology of nonunion to develop new minimally invasive therapeutics for bone reconstruction that do not present the side effects of conventional surgical techniques while achieving equivalent benefits. Proteomics is an interesting approach to search for new protein involved in nonunion pathology. Surface-enhanced laser desorption/ionization time of flight mass spectrometry (SELDI-TOF–MS) and two-dimensional difference gel electrophoresis (2D-DIGE) techniques are both complementary for biomarker search, investigating 1–30 and 20–200 kDa protein mass range, respectively. SELDI ProteinChip array surfaces include a set of classic chromatographic chemistries for capturing proteins according to their physico-chemical properties and displaying various protein profiles from an individual biological sample [11]. The principle of 2D-DIGE comparative proteomic analyses relies on the separation of proteins by 2D gel electrophoresis and comparison of proteins levels using different fluorescent dyes [12]. For both methods, high or low abundance protein signals are flagged for consideration as potential biomarkers of nonunion disease, while proteins in serum that give similar signals in nonunion and in controls are ignored. Our goal with the present study was to identify biomarkers associated with non-healing bone fractures in a nonunion atrophic population using an exploratory, observational, retrospective, cohort study design. The discovery of a set of new mediators could serve as a bridge between clinical and laboratory procedures and promote a better understanding of the physiopathology of bone healing.

## Methods

### Healthy volunteers (n = 35) and nonunion patients (n = 33)

Thirty-three atrophic nonunion (NU) patients were recruited through hospital outpatient clinics. All atrophic nonunion patients fulfilled established diagnostic criteria as described in [13] and failed to demonstrate any radiographic improvement for 6 consecutive months. Thirty-five healthy volunteers (HV) served as the control group. Epidemiologic data of HV and NU patients are summarized in Table 1. All subjects were qualified for entry into the study considering the following exclusion criteria: septic nonunion, head injury, diseases that might interfere with bone metabolism such as renal insufficiency, liver diseases, systemic inflammation (CRP > 0.5 mg/dL), osteoporosis and malabsorption. The study protocol was approved by the local institutional review boards of CHU Hospital of Liège (Research Ethics Committee—human protocol #2005-020—Principal Investigator: Prof M. Malaise). The bone sites of nonunion were: 7 radius, 5 humerus, 2 fibula, 2 scaphoids, 3 ulna, 3 metatarsus, 1 iliac bone, 3 clavicles, 2 femur and 5 tibia. The mean time (±standard deviation—SD) from the fracture related injury was 19 ± 9 months. All HV did not experience any fracture except two who broke a small bone and reconsolidated correctly 1 year prior to the inclusion in the study. All patients in the nonunion groups were on non-steroid anti-inflammatory drugs and analgesics such paracetamol (at a dose <4 g/day) or tramadol (at a dose <150 mg/day). One-third of the patients were taking anxiolytics such as alprazolam (at a dose <1 mg/day). HV were not taking any treatment.

**Table 1:** Demographic and clinical characteristics of healthy control and nonunion patients used in the study

	HV	NU	P values
n	35	33	
Age [median (range)]	32 (23–78)	44 (18–78)	0.004
Sexe (% of female)	62 %	31 %	0.003
BMI [median (range)]	26.1 (20.5–31.1)	26.8 (18.8–37.1)	NS
Smokers	28 %	58 %	0.03
Daily alcohol	0 %	29 %	0.003
*Comorbidities*			
Fracture history in the past year of visit	6 %	100 %	<0.001
Rheumatic diseases (osteoarthritis, psoriatic arthritis and rheumatoid arthritis)	0 %	14 %	NS
Gastric (heartburn and esophagitis)	0 %	14 %	NS
Diabetes (NIDDM)	0 %	10 %	NS
Pulmonary (asthma, emphysema, chronic pulmonary disease, pneumothorax)	0 %	24 %	0.006
Cardiovascular (ischaemia, deep vein thrombosis, infarction)	0 %	14 %	NS
High blood pressure	0 %	17 %	NS
Hypercholesterolemia	0 %	17 %	NS
Hypothyroidism	0 %	0 %	NS
Hypovitamin D	0 %	3 %	NS
*Treatment*			
NSAID and/or analgesic and/or tramadol	0 %	100 %	<0.001
Anxiolytics	0 %	33 %	<0.001

P value (quantitative data): Mann-Whitney test

P value (qualitative data): Yates’continuity corrected χ^2^

*HV* healthy volunteers; *NU* nonunions; *NIDDM* noninsulin-dependent diabetes mellitus

### Serum processing

Blood samples were allowed to coagulate in plain glass tubes during 30–60 min. Serum was obtained by centrifugation at 2800 rpm for 10 min, aliquoted and immediately frozen at −80 °C until required for proteomics analysis.

### Proteominer®

The use of hexapeptide libraries (ProteoMiner^®^, Biorad Laboratories Inc., Hercules, CA, USA) can significantly increase the detection of medium- and low-abundance proteins by the partial removal of high abundance proteins [14]. Proteominer® treatment was therefore applied to the entire cohort of serum samples. All serum samples were processed in 5 days according to manufacturer’s instructions. After removal of unbound fraction with PBS buffer (3 × 5 min of incubation), proteins were eluted with 3 × 100 µl of 25 mM Tris, 7 M urea, 2 M thiourea, 4 % CHAPS. A quality control (serum provided from a healthy volunteer) was run throughout the 5 days of Proteominer® process to control the reproducibility across the time. It is presented in Additional file 1: Appendix Figure 1.

### SELDI-TOF–MS

The 68 serum samples were loaded in duplicates as crude serum samples or Proteominer® eluate on two types of ProteinChip arrays: an anionic array (CM10) and an immobilized metal affinity capture bound with Cu^2+^ (IMAC-Cu) in order to capture a larger range of potential biomarkers. Four experimental conditions were therefore used in this study: (1) crude serum on CM10 arrays (pH9); (2) crude serum on IMAC-Cu^2+^ arrays; (3) Proteominer® eluate on CM10 (pH9) and (4) Proteominer® eluate on IMAC-Cu^2+^ arrays.

Crude serum samples were first denatured within 1.5 volume of 7 M urea, 2 M thiourea, 2 % CHAPS in 50 mM Tris buffer, and then diluted in six volumes of the following binding buffers: 100 mM Tris, pH9 (for CM10 arrays) or 100 mM phosphate buffer, 0.5 M NaCl, pH 7.6 (for IMAC-Cu^2+^ arrays). Proteominer® eluates were tenfold diluted in their corresponding binding buffers (see crude serum).

ProteinChip arrays were prepared according to manufacturer’s instructions and as previously described in [15]. Briefly, IMAC-Cu^2+^ arrays were successively incubated with 2 × 10 µL of CuSO_4_, rinsed with H_2_O, then incubated with 0.1 M acetate buffer (pH4) and finally rinsed with H_2_O. Then, CM10 and IMAC-Cu^2+^ arrays were equilibrated with 2 × 10 µL of binding buffer for 5 min. Ten microliters of each diluted serum (crude or eluate) mixture was applied, in duplicate, on the ProteinChip array and incubated for 1 h at room temperature. After discarding any remaining sample, arrays were washed three times with 10 µL of their respective binding buffer for 5 min, and then briefly rinsed with distilled water. Chips were air-dried and stored in the dark at room temperature until used for SELDI-TOF-MS analysis. A matrix solution [α-cyano-4-hydroxycinnamic acid (CHCA)] (Ciphergen Biosystems) was prepared according to the manufacturer’s instructions in 50 % volume/volume ACN and 0.5 % TFA. Before SELDI-TOF-MS analysis, 1 µL of 1:2 dilution of saturated CHCA solution was applied onto each spot and allowed to air dry. Chips were read on a PCS4000 ProteinChip reader in an *m/z* (mass to charge ratio) of 1100–30,000. Chips corresponding to the 68 serum samples were read over the day of their preparation to limit variability across the time. One day was used per experimental condition leading to four SELDI-TOF-MS experimental days. Complementarity observed between the four selected conditions is illustrated in Additional file 2: Appendix Figure 2. Standardization of experimental conditions was carried out in an effort to minimize the effects of irrelevant sources of fluctuation. Serum samples were applied randomly in order to avoid any artefact due to experimental handling. Replicates were not applied on the same chip array.

Mass accuracy was calibrated externally using the All-in-1 Peptide Standard (Ciphergen Biosystems) complemented with cytochrome c (MW: 12360) and myoglobin (MW 16951.5). Calibration was carried out according to the manufacturer’s instructions and is illustrated in Additional file 3: Appendix Figure 3. Pre-processing of spectra involving calibration, baseline subtraction, noise calculation, spectra alignment and total ion current normalization were completed before statistical analysis. Coefficients of normalization were in the range of 0.7–1.5. Peak detection was performed using ProteinChip Biomarker Wizard software 3.0 (BioRad).

### 2D-difference gel electrophoresis (2D-DIGE) analysis

Eleven pools of Proteominer® eluate were constituted inside both group, HV and NU, and were separated by 2D-DIGE. Protein content was determined using PlusONE 2-D Quant Kit (GE Healthcare, Uppsala, Sweden).

#### Analytical gels

Twenty-five µg of proteins from each pool were labelled separately in duplicate with 0.2 nmol of Cy3 or Cy5 dyes (GE Healthcare, Diegem, Belgium) for an incubation time of 30 min. Internal standard was obtained by pooling equal amounts of proteins (25 µg) of each biological sample and labelled with Cy2 (GE Healthcare, Diegem, Belgium). Following 30 min of incubation in darkness, the labelled samples were quenched with additional 0.2 µL of 10 mM Lysine (Sigma-Aldrich, Schnelldorf, Germany) and submitted to another 10 min incubation in darkness. Experimental 2D-gel electrophoresis, image acquisition and analysis, and PMF/MS–MS protein identification protocols were already described in a previous study [16]. Briefly, pairs of randomly chosen Cy3 and Cy5 samples were mixed and pooled with 25 µg of Cy2-labeled internal standard for 2D-DIGE experiments. Gels were scanned using a Typhoon 9400 Laser scanner (GE Healthcare, Piscataway, NJ, USA).

#### Preparative gels

For protein identification, two preparative gels were loaded with 250 µg of unlabeled proteins from either serum samples of HV and NU patients after Proteominer® processing and with 25 µg of the internal standard. Gels were run under the same conditions as analytical gels. Spots of interest were excised using an Ettan Spotpicker robot (GE Healthcare, Piscataway, NJ, USA). Proteins were digested with 20 ng/L of trypsin (Roche, porcine, proteomic grade) for 4 h at 37 °C using a Janus Robot (Perkin Elmer, Waltham, MA, USA). Resulting peptides were extracted and rehydrated in 10 µL of formic acid (1 %). Automated spectra acquisition was performed using an Ultraflex II MALDI mass spectrometer (Bruker Daltonics, Billerica, MA). Peptides identification was managed using Biotools v.3.1.software (Bruker Daltonics, Billerica, MA) with an in-house hosted Mascot v2.2.2 server. Human taxonomy was used for database search with 100-ppm mass accuracy. Identification was significant for peptide mass fingerprint with a P < 0.05 and a Mascot protein score ≥70.

### Quantification of hepcidin-25 in serum samples by LC–MS/MS

Hepcidin-25 levels of serum samples (35 HV and 33 NU) were measured by a LC-chip coupled to a nanoelectrospray/ion trap/MS operating in positive mode. Extraction procedure, calibration standard preparation, chromatographic and MS parameters were previously described by Houbart et al. [17]. Briefly, serum samples were thawed at room temperature and centrifuged during 10 min at 13,400 rpm. Fifty microlitre of each serum sample were added to as solution containing 50 μL of 5 % phosphoric acid, 12.5 μL internal standard solution to reach a concentration of 10 ng/mL and 12.5 μL of hepcidin solution or water, depending of the type of sample (standard or blank sample, respectively). A Waters Oasis μElution WCX 96 well plate was used to prepare the samples for analysis. The sorbent was first conditioned by 300 μL methanol followed by 300 μL water. The entire sample was then transferred in the well and drawn through the sorbent with a vacuum manifold. The plate was then washed with 200 μL of 25 mM ammonium acetate pH6.8, 200 μL of H_2_O/ACN (60:40, v/v/) performed twice. The extracts were eluted with 2 × 50 μL of ACN/H_2_O/TFA (75:25:1, v/v/v). The eluates were then evaporated in a vacuum evaporator and reconstituted in 100 μL ACN/H_2_O/TFA (33.8:66.2:0.1, v/v/v).

### Immunoprecipitation (hepcidin-25)

Fifteen µg of anti-hepcidin-25 (ab30760; Abcam, Cambridge, UK) antibodies were coupled with 50 µL of protein G + beads overnight at 4 °C. Twenty microlitre of serum provided by a NU patient was then incubated with beads for 2 h at 4 °C. After several washes with PBS containing 0.1 % of Tween-20, bound fractions were eluted with 100 mM acetic acid containing 30 % acetonitrile. Unbound (Flowthrough) and bound (Eluate) fractions were analyzed on IMAC-Cu^2+^ arrays by SELDI-TOF-MS. Non-specific IgG antibodies were used as negative controls.

### Western-blotting (complement C6 and S100A9)

Complement C6 and S100A9 proteins were assessed by Western blot. Briefly, 2 µL of serum was run on 4–12 % NUPAGE Bis–Tris polyacrylamide gels (Invitrogen), transferred and incubated with anti-C6 monoclonal antibody (dilution 1/2000; ab71942; Abcam) or with anti-S100A9 polyclonal antibody (dilution 1/1000; sc20173; Santa Cruz). We then incubated with a mouse or a rabbit secondary antibody (dilution 1/5000; Dako) to detect C6 or S100A9, respectively. Proteins were revealed with an enhanced chemiluminescence detection method according to the manufacturer’s instructions (GE Healthcare). Band intensities were quantified by ImageQuant LAS 4000 software (GE Healthcare) and are expressed as pixel counts representing integrated signal intensities.

### Enzyme-linked immunosorbent assay

A commercially available sandwich ELISA was used for apolipoprotein E (ApoE) quantification in serum according to manufacturer’s instructions (Catalog# DAPE00, R&D Systems).

### Nephelometry

Serum levels of complement C3 and C4 were determined by an immunonephelometric method on a BNII nephelometer (Dade Behring/Siemens) with specific antibodies (Siemens, Marburg, Germany).

### Statistics

#### Demographic statistics

For quantitative data, differences between groups were analyzed using a Mann–Whitney U test. For qualitative data, we used the Yates’ continuity corrected χ^2^ test. P < 0.05 were considered as statistically significant.

#### Statistical analysis for SELDI-TOF–MS data

Peak intensities were analysed by two statistical approaches, a nonparametric Mann–Whitney U tests and a machine-learning algorithm called extra-trees [18]. Extra-trees is a decision-tree multivariate analysis that estimates the relevance or relative contribution of each peak to the classification of 2 groups [18]. The latter approach allows *m/z* values to be ranked according to their relevance for differentiating NU from HV groups based on quantitative estimates of the percentage of information (% of info) supplied (see Additional file 4: Appendix Table 1). P < 0.05 were considered as statistically significant. Q values were calculated using FDR (1000 permutations) for multiple testing. The most important biomarkers were corrected for age, gender, smoking status, consumption of alcohol and the presence of pulmonary diseases by a multivariate analysis performed on the SAS software version 9.4.

#### Statistical analysis for 2D-DIGE

Two dimensional-gel images were analysed using Decyder 2D differential analysis software (v.6.5, GE Healthcare). The differentially expressed proteins in HV vs NU were detected using biological variance analysis (BVA) module. The unpaired student’s *t* test was used and a P < 0.05 was considered as statistically significant. Protein spots that showed a significant fold change of at least 1.2 with a P < 0.05 (unpaired student’s *t* test) in HV vs NU comparison were submitted to identification.

#### Statistical analysis for biomarkers validation

Statistical analysis was performed by GraphPad Prism software. Protein levels for hepcidin, complement C6, S100A9, ApoE, complement C3 and complement C4 in serum of HV and NU patients were compared using the non-parametric Mann–Whitney test and were expressed as median and interquartile range. P < 0.05 were considered as statistically significant. P values were also compared to the following significance after Bonferroni correction: α = 0.05/6 = 0.008.

## Results

### SELDI-TOF–MS analysis

SELDI-TOF–MS was used to provide serum protein profiles ranging from 1 to 30 kDa for each HV and NU patients. Serum samples were applied on ProteinChip arrays as crude serum samples but also after equalization (removal) of the most abundant proteins (Proteominer® kit) for the detection of medium- and low-abundance proteins. For the 35 HV and 33 NU, crude serum samples and Proteominer® eluates were run in duplicate. 136 spectra were collected on CM10 and IMAC-Cu^2+^ ProteinChip arrays for each of the four experimental conditions: (1) crude serum on CM10 arrays (pH9); (2) crude serum on IMAC-Cu^2+^ arrays; (3) Proteominer® eluate on CM10 (pH9) and (4) Proteominer® eluate on IMAC-Cu^2+^ arrays (Additional file 2: Appendix Figure 2). Biomarker Wizard software resolved 157, 188, 167 and 153 peaks for experimental conditions 1–4, respectively. Peak intensities in the HV group were compared to corresponding peak intensities in the NU group. Peaks of similar intensities were ignored when peaks of different intensities were of particular interest. Peaks were then classified according to their percentage of importance (% imp), given by the extra-trees multivariate analysis, in differentiating HV vs NU spectra. A P value and a Q value for each biomarker were also calculated. The most relevant biomarkers for the four conditions are summarized in Additional file 4: Appendix Table 1.

### Identification of potential SELDI biomarkers

**Fig. 1:**
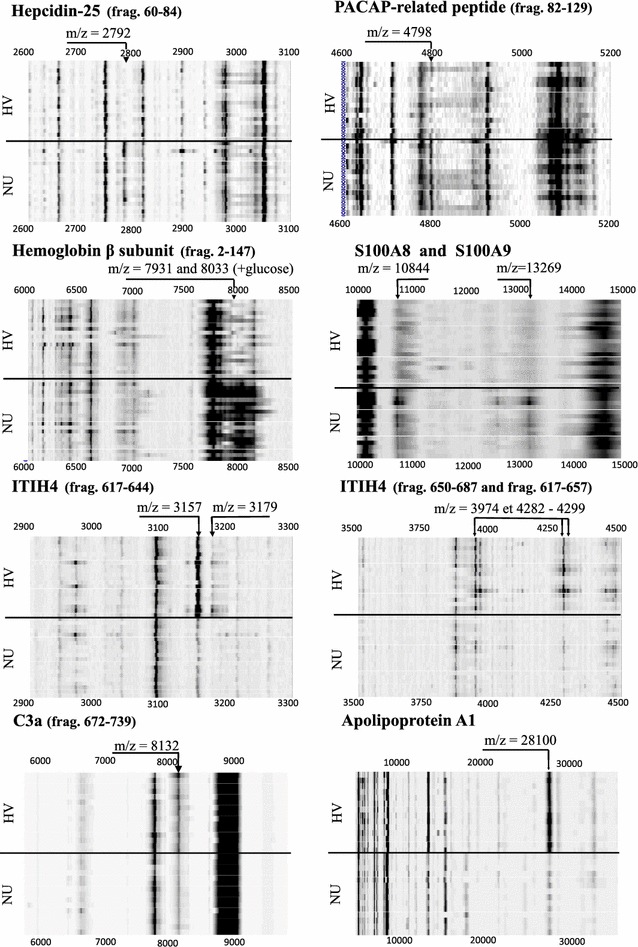
SELDI-TOF–MS gel view spectra for samples collected from at least 15 healthy controls and 15 nonunion patients. Illustration of identified biomarkers: hepcidin-25, pituitary adenylate cyclase-activating polypeptide (PACAP)—related peptide, hemoglobin β subunit, S100A8, S100A9* variant, S100A9, inter-α-trypsin inhibitor heavy chain H4 (ITIH4), complement factor C3a and apolipoprotein A1. *HV* healthy volunteers; *NU* nonunion patients

Table 2 summarizes selected biomarkers that have been identified. Figure 1 illustrates these biomarkers in a SELDI-TOF–MS gel view spectra collected from at least 15 HV and 15 NU patients.

**Table 2:** Characterization of SELDI-TOF-MS biomarkers: P values were obtained according to the non-parametric Mann–Whitney U test (see also Additional file 4: Appendix Table 1)

m/z	MW	ID	Amino acid sequence	Regulation			
				HV vs. NU	P value	Q value	Adj. P value
Hepcidin (P81172)							
2191	2199	Hepcidin-20 (frag 65–84)	ICIFCCGCCHRSKCGMCCKT	Up	<0.001	0.007	0.023
2792	2797	Hepcidin-25 (frag 60–84)	DTHFPICIFCCGCCHRSKCGMCCKT	Up	<0.001	0.004	0.023
PACAP-related peptide (P18509)							
4798	4800	PRP-48 (frag 82–129)	DVAHGILNEAYRKVLDQLSAGKHLQSLVARGVGGSLGGGAGDDAEPLS	Up	<0.001	0.021	0.037
Hemoglobin β subunit (P68871)							
15866	15867	Hemoglobin β subunit (frag 2–147)	VHLTPEEKSAVTALWGKVNVDEVGGEALGRLLVVYPWTQRFFESF GDLSTPDAVMGNPKVKAHGKKVLGAFSDGLAHLDNLKGTFATLSE LHCDKLHVDPENFRLLGNVLVCVLAHHFGKEFTPPVQAAYQKVVA GVANALAHKYH	Up	0.021	NS	NS
7931	15867	Hemoglobin β subunit (frag 2–147) [2H+]	VHLTPEEKSAVTALWGKVNVDEVGGEALGRLLVVYPWTQRFFESF GDLSTPDAVMGNPKVKAHGKKVLGAFSDGLAHLDNLKGTFATLSE LHCDKLHVDPENFRLLGNVLVCVLAHHFGKEFTPPVQAAYQKVVA GVANALAHKYH	Up	<0.001	0.020	0.027
S100A8 (P05109) and S100A9 (P06702)							
10844	10835	S100A8 (frag 1–93)	MLTELEKALNSIIDVYHKYSLIKGNFHAVYRDDLKKLLETECPQYIR KKGADVWFKELDINTDGAVNFQEFLILVIKMGVAAHKKSHEESHKE	Up	<0.001	0.008	0.0002
5419	10835	S100A8 (frag 1–93) [2H+]	MLTELEKALNSIIDVYHKYSLIKGNFHAVYRDDLKKLLETECPQYIR KKGADVWFKELDINTDGAVNFQEFLILVIKMGVAAHKKSHEESHKE	Up	<0.001	0.022	
13269	13274	S100A9 (frag 1–114) [oxydized form]	MTCKMSQLERNIETIINTFHQYSVKLGHPDTLNQGEFKELVRKDLQ NFLKKENKNEKVIEHIMEDLDTNADKQLSFEEFIMLMARLTWASH EKMHEGDEGPGHHHKPGLGEGTP	Up	<0.001	0.004	0.0024
6346	12691	S100A9* (frag 5–114) [2H+] [Ser-actelyl]	S(ac)QLERNIETIINTFHQYSVKLGHPDTLNQGEFKELVRKDLQ NFLKKENKNEKVIEHIMEDLDTNADKQLSFEEFIMLMARLTWASH EKMHEGDEGPGHHHKPGLGEGTP	Up	<0.001	0.013	
Inter-alpha-trypsin inhibitor H4 (ITIH4) (Q14624)							
3157	3157	ITIH4 (isoform 1) frag 617–644	NVHSGSTFFKYYLQGAKIPKPEASFSPR	Down	<0.001	0.007	0.017
3179	3180	ITIH4 (isoform 1) frag 617–644 [aduct Na+]	NVHSGSTFFKYYLQGAKIPKPEASFSPR	Down	<0.001	0.007	0.017
3974	3972	ITIH4 (isoform 1) frag 650–687	QAGAAGSRMNFRPGVL SSRQLGLPGPPDVPDHAAYHPF	Down	<0.001	0.020	
4282	4282	ITIH4 (isoform 2) frag 617–657	NVHSAGAAGSRMNFRPGVLSSRQLGLPGPPDVPDHAAYHPF	Down	<0.001	0.015	
4299	4298	ITIH4 (isoform 2) frag 617–657 [Met.Ox]	NVHSAGAAGSRM(ox)NFRPGVLSSRQLGLPGPPDVPDHAAYHPF	Down	<0.001	0.015	
Complement C3 alpha chain (P01024)							
8132	8132	C3a (frag 672–739)	SVQLTEKRMDKVGKYPKELRKCCEDGMRENPMRFSCQRRTRFISLG EACKKVFLDCCNYITELRRQHA	Down	<0.001	0.022	NS
Apolipoprotein A–I (P02647)							
28106	28061	ApoA1 (frag 25–267)	DEPPQSPWDRVKDLATVYVDVLKDSGRDYVSQFEGSALGKQLNLKL	Down	<0.001	0.029	NS
28099			LDNWDSVTST FSKLREQLGPVTQEFWDNLEKETEGLRQEMSKDLEE VKAKVQPYLDDFQK KWQEEMELYRQKVEPLRAELQEGARQKLHEL QEKLSPLGEEMRDRARAHVDALRTHLAPY SDELRQRLAARLEALKEN GGARLAEYHAKATEHLSTLSEKAKPALEDLRQGLLPVLESFKVSFLSALEEYTKKLNT	Down	<0.001	0.05	

Associated Q values were calculated using FDR (1000 permutations) for multiple testing. Adjusted P value for age, gender, smoking status, consumption of alcohol and the presence of pulmonary diseases were performed by multivariate analysis on the SAS software version 9.4

*m/z* mass/charge ratio of the peak detected by SELDI-TOF-MS, *MW* molecular weight of the related protein or peptide, *ID* identification of the related protein or peptide, *NS* not significant

**Fig. 2:**
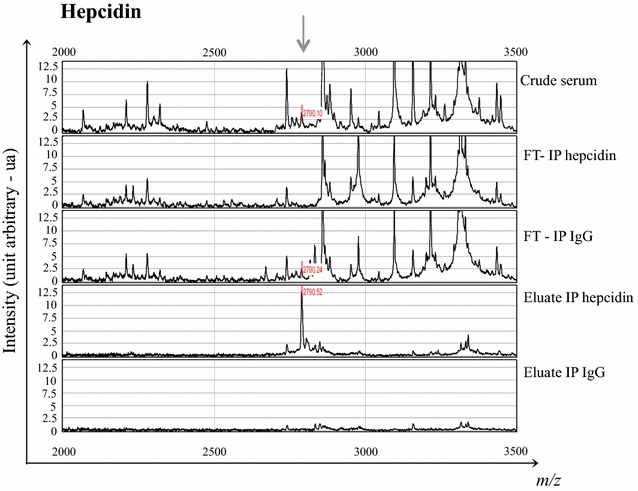
Immunodepletion for hepcidin identification. Purification from serum and identification of the protein fragment at *m/z* value of 2792 as hepcidin-25. Identification by depletion of serum using specific and non specific antibodies followed by elution from the immune complexes. *FT* flow-through; *IP* immunoprecipitation

Direct protein identification cannot be achieved by SELDI-TOF–MS technology as opposed to other MS/MS approaches. In a first attempt, protein identification was performed by introducing sought *m/z* values in Tagident website (http://www.web.expasy.org/tagident) and therefore getting access to related protein identification. Accordingly, *m/z* values 2191, 2792, 4798 and 15,866 were introduced in Tagident. Search for protein identification was run in a molecular weight range of 0.5, 0.5, 0.1 and 0.01 %, respectively. Hepcidin-20, hepcidin-25, PACAP-related peptide (PRP-48) and hemoglobin β subunit were detected as potential related proteins (Table 2; Fig. 1). Identification was confirmed for hepcidin-25 (Fig. 2) by immunodepletion using specific antibodies (Fig. 2). Peaks of interest were depleted after immunodepletion (see FT-IP hepcidin in Fig. 2). Proteins were then eluted from their immune complex and applied on ProteinChip arrays to be analysed by SELDI-TOF-MS (see eluate IP hepcidin in Fig. 2). The depletion was only visible with specific anti-hepcidin-25 and not with negative control antibodies. A similar experiment was conducted previously to confirm the identification of hemoglobin β subunit [19]. Peaks at *m/z* 7931 (Fig. 1) and 15,866 were the doubly (2H+) and singly (1H+) charged forms of hemoglobin β subunit (Table 2). Interestingly, glycated forms of hemoglobin β subunit, previously characterized with MALDI-TOF by Lapolla et al. [20, 21], could also be detected on spectra of atrophic patients. Protonated molecules produced by the condensation of one glucose molecule on the hemoglobin β subunit could be observed at *m/z* 8033 (Fig. 1) and 16,022 for the doubly (2H+) and singly (1H+) charged forms, respectively (Additional file 4: Appendix Table 1).

In previous publications, we have extensively described the identification of S100A8, S100A9 and its isoform S100A9* proteins after proteomic analysis by SELDI-TOF-MS [15, 22]. Our longstanding experience with these 3 proteins allowed us to confirm their presence in the NU cohort. Peaks at *m/z* 5419 and 10,844 (Fig. 1) were the doubly (2H+) and singly (1H+) charged forms of S100A8 (Table 2). Peak at *m/z* 6346 was the doubly (2H+) charged forms of S100A9*. S100A9* variant results from a translation beginning at amino acid residue 5 and acetylation of amino acid residue 6 (Ser residue), yielding a calculated mass of 12,691 Da. Mass to charge value 13,269 (Fig. 1) corresponds to the oxidized form of S100A9 (Table 2). Other groups [23–27] previously identified some biomarkers that were also found in our work such as inter-alpha-trypsin inhibitor H4 (ITIH4) fragments, complement C3α-chain fragment and apolipoprotein A-I (ApoA1) protein after SELDI-TOF-MS proteomic analysis. Indeed, *m/z* values 3157, 3179 and 3974 was characterized as ITIH4 (isoform 1) fragment 617–644 [26], ITIH4 (isoform 1) fragment 617–644 [adduct Na^+^] [26] and ITIH4 (isoform 1) fragment 650–687 [23, 26] (Table 2; Fig. 1). Mass to charge values 4282 and 4299 represent ITIH4 (isoform 2) fragment 617–657 with and without methionine oxidation [23, 24]. Similarly, *m/z* value 8132 was identified as a C-terminal end truncated form of complement C3a [25] (Table 2). Finally, ApoA1 protein was related to *m/z* value 28.1 kDa [27] (Table 2; Fig. 1). P values, Q values and adjusted P values are summarized in Table 2. After correction, it appears that all adjusted P-values for age, gender, smoking status, consumption of alcohol and the presence of pulmonary diseases remained statistically significant except for C3a and ApoA1 that need therefore careful consideration.

### 2D-DIGE

**Fig. 3:**
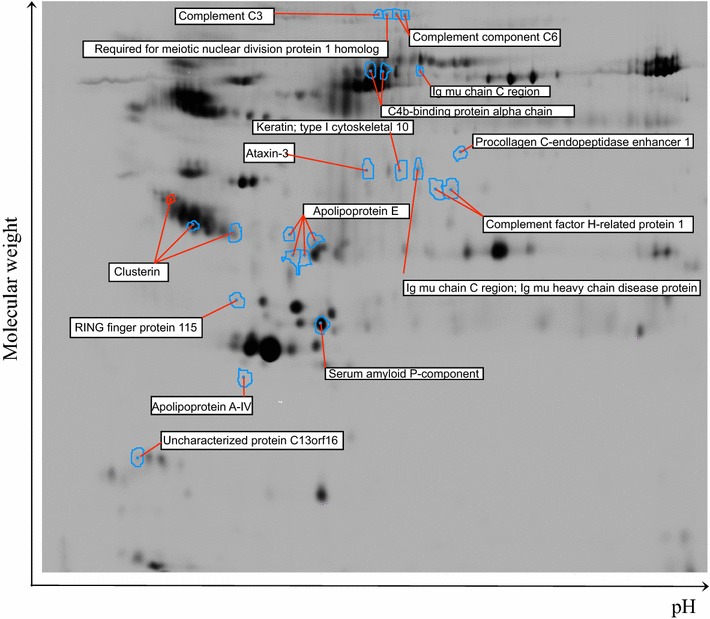
Gel master and location of the protein spots picked: contours are protein spots with at least 1.2-fold expression change (P < 0.05, student t-test) and proteins significantly identified with the Ultraflex II MALDI mass spectrometer and Biotools v.3.1.software with an in-house hosted Mascot v2.2.2 server

**Fig. 4:**
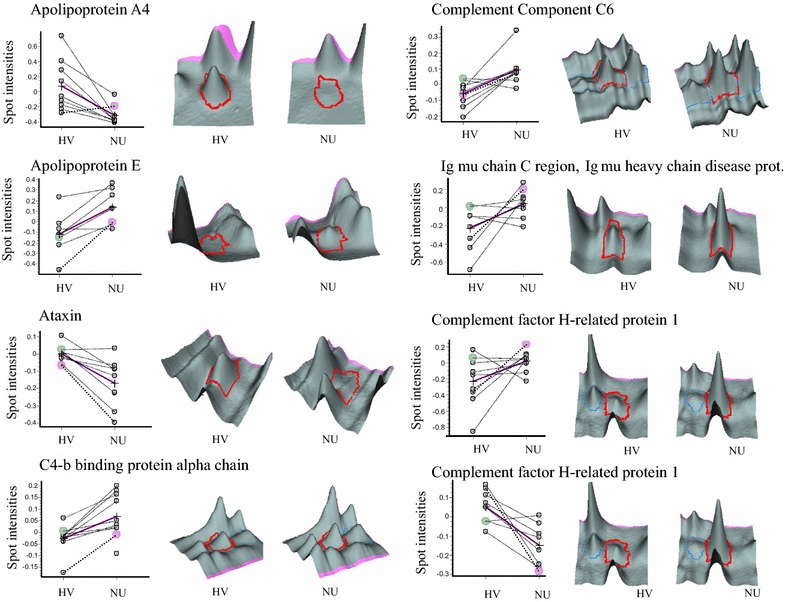
Comparative analysis of selected protein spot intensities with the BVA module of the Decyder 6.5 software. Spot intensities obtained for each pool of healthy volunteers (*HV*) and nonunion (*NU*) patients were plotted on a graph. 3-D images represent the 3D view of selected spots obtained from one representative HV and NU pool for each biomarker of interest

2D-DIGE was used to provide serum protein profiles ranging from 20 to 200 kDa. Eleven pools of Proteominer® eluate were constituted inside both group, HV and NU, and were separated by 2D-DIGE. Figure 3 illustrates one representative 2D-DIGE gel view of NU-serum samples after Proteominer® processing. From analytical gels, about 4500 spots were detected over the selected pH range (pH 3–11) and 24 spots were differentially expressed in NU compared to HV controls with a P value <0.05 (Table 3). Twenty spots were up-regulated in NU whereas four were down-regulated. These biomarkers are summarized in Table 3 including their identification, statistical analysis (p value, *t* test), fold-change in expression levels, isoelectric point (pI), molecular weights (MW) and Mascott score values obtained by MALDI (Peptide mass fingerprinting (PMF) or MS/MS). Figure 4 represents semi quantitative graph of spot intensities obtained for each pooled samples. The 3D view is the representation of one HV and NU pool.

**Table 3:** Proteins with at least 1.2-fold expression change (P < 0.05, student t test) in serum of nonunion patients identified with the Ultraflex II MALDI mass spectrometer and Biotools v.3.l.software with an in-house hosted Mascot v2.2.2 server

No	Master no	Protein ID	Name	P value	Fold	pI	MW	Identification	Score Mascott
1	2951	APOA4_HUMAN	Apolipoprotein A–IV	0.005	−3.27	5.28	45371	PMF	78
2	2314	APOE_HUMAN	Apolipoprotein E	0.031	1.73	5.65	36246	PMF	93
3	2296	APOE_HUMAN	Apolipoprotein E	0.036	1.65	5.65	36246	MS/MS	34
4	2218	APOE_HUMAN	Apolipoprotein E	0.037	1.42	5.65	36246	PMF	86
5	2190	APOE_HUMAN	Apolipoprotein E	0.028	1.35	5.65	36246	PMF	133
6	1757	ATX3_HUMAN	Ataxin-3	0.011	−1.4	4.81	42097	PMF	57
7	961	C4BPA_HUMAN	C4b-binding protein alpha chain	0.0043	1.31	7.15	69042	PMF and MS/MS	72 and 44
8	956	C4BPA_HUMAN	C4b-binding protein alpha chain	0.034	1.29	7.15	69042	PMF and MS/MS	111 and 62
9	2200	CLUS_HUMAN	Clusterin	0.018	1.39	5.89	53031	PMF and MS/MS	70 and 63
10	2131	CLUS_HUMAN	Clusterin	0.02	1.22	5.89	53031	PMF and MS/MS	73and101
11	1946	CLUS_HUMAN	Clusterin	0.024	1.28	5.89	53031	MS/MS	17
12	3896	CM016_HUMAN	Uncharacterized protein C13orf16	0.0079	1.37	4.99	16981	PMF	57
13	432	co3_human	Complement C3	0.016	1.25	6.02	188569	PMF	119
14	444	co6_human	Complement component C6	0.0029	1.44	6.39	108367	PMF and MS/MS	88 and 17
15	421	co6_human	Complement component C6	0.032	1.27	6.37	108369	PMF	76
16	1883	FHR1_HUMAN	Complement factor H-related protein 1	0.0012	−1.58	7.38	38766	PMF and MS/MS	55 and 31
17	1874	FHR1_HUMAN	Complement factor H-related protein 1	0.04	1.54	7.38	39766	PMF	85
18	974	IGHM_HUM	lg mu chain C region	0.018	1.22	6.35	49960	PMF	63
19	1766	IGHM_HUMAN. MUCB_HUMAN.	lg mu chain C region. lg mu heavy chain disease protein	0.021	1.75	6.35	49960	MS/MS	34
20	1765	K1C10_HUMAN	Keratin. Type I cytoskeletal10	0.024	−1.43	5.13	59020	PMF	57
21	1619	PCOC1_HUMAN	Procollagen C-endopeptidase enhancer 1	0.05	1.25	7.41	48797	PMF	61
22	427	RMND1_HUMAN	Required for meiotic nuclear division protein 1 homolog	0.0023	1.34	8.88	51970	PMF	56
23	2528	RN115_HUMAN	RING finger protein 115	0.043	1.21	5.39	34252	PMF	60
24	2660	SAMP_HUMAN	Serum amyloid P-component	0.017	1.25	6.1	25485	PMF	87

Crude serum samples cannot be directly applied on 2D-gel due to the presence of highly abundant proteins (albumin and IgG) masking medium to low-abundance protein signals. Accordingly, Proteominer® pre-processing was mandatory for digging deeper into the proteome. Nevertheless, it was also responsible for protein level equalization, therefore limiting fold change variation from 1.2 to 3.5-fold when comparing NU to HV groups. However, validation of some biomarkers by alternative approach described below (Western blotting, ELISA and others) confirmed the robustness of our results and allowed a better visualization of protein levels in serum before pre-processing steps.

### Protein validation

**Fig. 5:**
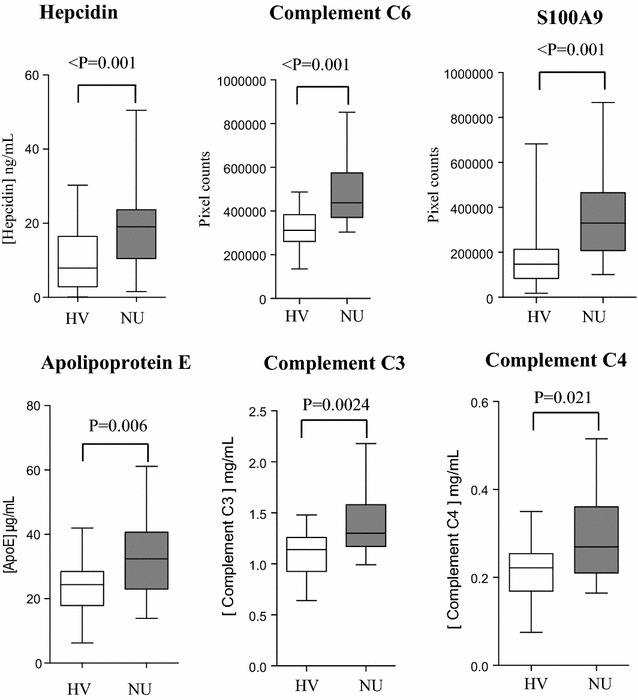
Validation of selected biomarkers by alternative approaches: Hepcidin (MS/MS quantification), complement C6 (western blotting), S100A9 (western blotting), apolipoprotein E (ELISA), Complement C3 (nephelometry) and Complement C4 (nephelometry). Statistical analysis by Mann–Whitney U test was performed to compare biomarkers intensity in serum of healthy volunteers (HV) *vs* nonunion (NU) patients. For western blotting, band intensities were quantified by ImageQuant LAS 4000 software and are expressed as pixel counts representing integrated signal intensities

Hepcidin quantification was performed by LC–MS/MS in serum samples of both groups and a higher expression level was observed in NU-serum samples compared to HV (P < 0.001) (Fig. 5).

Western blot analysis with specific antibodies was used to validate expression changes of two proteins: complement C6 and S100A9. The integrated signal intensities of specific immunoreactive band was quantified for each protein, and intensities were compared between NU and HV groups. Both proteins showed an increased expression level in NU compared to HV (P < 0.001). Apolipoprotein E was quantified using ELISA kit and elevated expression level was observed in NU compared to HV (P = 0.006). Finally, C3 and C4 complement factors were quantified by nephelometry and their expression levels were significantly higher in NU compared to HV (0.0024 and 0.021, respectively). All biomarkers remained statistically significant after Bonferroni correction when comparing intensities in HV vs. NU groups, except for C4 complement.

## Discussion

Bone formation is controlled by many factors acting locally to regulate proliferation, differentiation and activity of osteoblasts. TGF-β and BMPs, as well as ligands of Wnt family are of course very well known effectors of bone formation. Several hormones, such as parathyroid hormone, steroids but also leptin can affect bone formation. The role played by leptin on bone remodelling illustrates the connection between lipid and bone metabolism [28]. To our knowledge, this is the first proteomics study investigating the simultaneous expression of serum proteins in patients with atrophic nonunion. Sera of nonunion patients and healthy volunteers were analysed by SELDI-TOF–MS and 2D-DIGE proteomics approaches using strict quality control criteria and optimized data acquisition and preprocessing parameters. Robust statistical approach by multivariate analysis was associated to the univariate approach for mass spectrometry data analysis to highlight potential makers. Several biomarkers were up-regulated (hepcidin, PACAP-related peptide, hemoglobin β subunit, S100A8, S100A9, ApoE and complement C3, C4 and C6) or down-regulated (ITIH4 fragments, C3a, ApoA1 and ApoA-IV) in serum of patients with atrophic fracture compared to controls. Some were validated according to alternative approaches and drew our specific attention. The differential serum proteome analysis of patients with atrophic fractures provided new insights on systemic protein expression that are linked to bone turn-over, inflammation, innate immunity, glycation and lipid metabolisms.

There are several limitations to this study. The results should be considered as preliminary given that the sample sizes were too small to draw definitive conclusions and an independent validation should be performed with a new cohort of freshly collected serum samples. Further, age, gender, smoking status, consumption of alcohol and the presence of pulmonary diseases were significantly divergent between HV and NU groups. However after correction, it appears that all adjusted P values remained statistically significant except for C3a and ApoA1 that need therefore careful consideration. Complement C4 was also not statistically significant after Bonferroni correction. Nevertheless, this study sheds some light on new markers that could slow down bone healing by enhancing a persistent and non-resolving inflammatory process.

Increased hepcidin levels were observed by SELDI-TOF-MS in patients with atrophic nonunion fracture compared to healthy volunteers, and were confirmed by quantitative MS/MS approach. Hepcidin is an iron-regulatory hormone that contributes to iron homoeostasis in human organism. In addition to having a role in iron regulation, hepcidin contributes to host defense and may modulate inflammatory processes [29]. It is mainly secreted by hepatocytes, but other tissues and cells express hepcidin including the brain, heart, kidney, adipose tissue, monocytes and macrophages [30–35]. Although iron is the main regulator of hepcidin in hepatocytes, inflammation appears to be the dominant regulator of hepcidin in monocytes and macrophages [29]. Incubation of the cultured monocytes with IL-6 increased hepcidin expression [36]. Similarly, hepcidin may also be induced in inflammatory condition (under IL-6 stimulation) in adipose tissue of obese patients [30]. In fracture, macrophages are mobilized at the fracture site to remove cell or matrix debris and to regulate tissue repair. Further, patients with atrophic nonunion have persistent elevation of the plasma inflammatory cytokines, CRP and IL-6 [2], and the latter could be responsible (in part) to the induction of hepcidin expression in monocytes/macrophages of those patients.

Hepcidin itself seems also closely associated with bone metabolism by enhancing osteoblastic differentiation of MSCs by activating BMP2/Smad and MAP/p38 pathways [37], but also by facilitating osteoclast differentiation [38]. If hepcidin appears regulated by inflammatory mediators, it could itself play a role in the osteoblast/osteoclast differentiation.

Complement pathway is also involved in inflammation and innate immunity. Patients with atrophic fracture showed higher levels of complement zymogens C3, C4 and C6 by 2D-DIGE whereas levels of the active form C3a were decreased in SELDI-TOF-MS experiments. Complement system is activated early after trauma in response to various stimuli to present a first line of defence of innate immunity against pathogen- and danger-associated molecular patterns (PAMPs and DAMPs). The role of complement to eliminate damaged cells is very well known but can be extended now to the regulation of cytokines network, coagulation cascade and even in bone repair [39, 40]. Indeed, hyper-activation of the complement cascade can lead to unfavourable outcome such as delayed fracture healing.

During the whole healing phase, complement factors are locally expressed to play a regulatory role dedicated to a constant cross talk between the bone and the immune system. Studies by Schraufstatter et al. and Ignatius et al. revealed that C3a and C5a were powerful chemokines for mediating MSC and osteoblasts migration [41, 42]. They also observed that C3 and C5 were secreted by undifferentiated and differentiated MSC and osteoblasts [43]. In our study, we observed a strong decrease of C3a levels in atrophic fracture compared to healthy conditions while levels of the related complement zymogen C3 were increased in nonunion patients compared to controls. Increased expression of C3 was also observed in serum of patients with femoral head osteonecrosis [44].

Another proteins family involved in immune response is S100 proteins. S100A8 and S100A9 proteins are calcium-binding proteins expressed in the cytoplasm of phagocytes. They belong to DAMP proteins acting as alarmins and triggering immune and inflammatory responses by interacting with receptors such as receptor for advanced glycation end products.

(RAGE) and toll-like receptors 4 (TLR4) [45, 46]. Increased S100A8 and S100A9 levels were observed by SELDI-TOF-MS in patients with atrophic nonunion fracture compared to healthy volunteers, and were confirmed by Western-blotting. S100A8 and S100A9 proteins are key players in the amplification of inflammatory reaction. They are highly up-regulated in various diseases, such as sepsis, rheumatoid arthritis, inflammatory bowel disease and cancer. Accordingly, these proteins are now well-recognized as biomarkers of inflammation. S100A8 and S100A9 are also associated with cartilage and bone [47]. Further, S100A8 produced by activated macrophages is also known to stimulate osteoclast differentiation and osteoclast function at the site of inflammation in experimental arthritis [48]. The authors mentioned that S100A8-stimulated osteoclasts contained significantly more actin rings per cell that create the acidic environment crucial for cathepsin K activity, and therefore suggested that S100A8 could enhance the bone resorptive capacity per osteoclast.

Diabetes is a well-known risk factor for delayed fracture healing, and elevated AGEs in blood may be a significant risk factor for diabetes. Increased expression of hemoglobin β-subunit was observed in serum of atrophic patients compared to healthy controls. Interestingly, glycated forms of hemoglobin β-subunit, previously characterized with MALDI-TOF by Lapolla et al. [20, 21], could also be detected on spectra of atrophic patients. Protein glycation level can provide effective indications of glycemic regulation and possible chronic complications in diabetes. Glycated hemoglobin (HbA_1c_) is one of the most powerful parameters that reflect average serum glucose concentration over a period of 4–8 weeks. HbA_1c_ is important for people with diabetes and is highly correlated with the risk of developing diabetes-related complications. One of the many organs affected by diabetes is bone. In diabetes, the state of the bone is altered, leading to an increased fracture risk and subsequently delay union, and potentially nonunion [49–52]. Although the association of hyperglycemia with bone healing complications has been well documented, little is known about glycated mediators that directly affect bone healing. AGEs in bone are associated with decreased systemic factors on osteoblast functions such as proliferation, differentiation and mineralisation but also increased apoptosis [53]. AGE-modified collagen is the most studied AGE product causing greater collagen stiffness. It should be now clarified if glycated hemoglobin β-subunit is playing a role in the non-healing bone fracture or if it is just a marker of AGE products.

Increased ApoE levels were observed by 2D-DIGE in patients with atrophic nonunion fracture compared to healthy volunteers, and were confirmed by ELISA. ApoE is the major protein component of several lipoprotein classes. It mediates the transport and clearance of lipids in plasma [54]. ApoE isoforms are involved in several cardiovascular and neurological pathologies [55], and recently, a new role for ApoE has emerged as a regulator of bone metabolism [56]. ApoE seems to be required for the sufficient uptake of vitamin K into osteoblasts and γ-carboxylation of osteocalcin, a marker of bone cell metabolism [56]. Alternatively, ApoE may function as a molecular switch that inhibits osteogenic and stimulates adipogenic differentiation of MSCs by inhibiting the canonical Wnt signalling [56]. Similarly, Bartelt et al. demonstrated that ApoE was involved in an inverse regulation of bone mass and fat mass in growing mice [57]. It remains that mechanisms by which ApoE regulates bone mass need to be clarified.

Finally, decreased ITIH4 levels were observed by SELDI-TOF-MS in atrophic nonunion fracture compared to healthy volunteers. The inter-α-trypsin inhibitors (ITI) are a family of structurally related plasma serine protease inhibitors involved in extracellular matrix stabilization by covalent linkage to hyaluronic acid [58]. They are secreted into the blood by the liver. The ITI family contains multiple proteins made up of a light chain and a variable number of heavy chains including H4. ITIH4 is present in plasma as a single-chain protein that contains a proline-rich region (PRR), which is readily cleaved by the plasma kallikrein giving rise to multiple small peptides [59]. ITIH4 fragmentation patterns is closely associated with different disease conditions [60] and is often increased in several cancer diseases such as breast or prostate cancer [26, 60]. However, fragmentation of ITIH4 is also decreased in some pathologies such as pancreatic cancer [24, 60], hepatic cirrhosis [61] and osteoporosis [23]. Our results are in adequacy with the decreased ITIH4 fragmentation pattern observed by Bhattacharyya et al. in patients with high bone turnover compared with the low/normal turnover controls. They hypothesized that the observed decrease of specific ITIH4 fragments was related to increased proteolysis, which is associated with the increased osteoclastic activity. However ITIH4 fragmentation seems to be more related to the proteolytic action of kallikrein in serum [59]. The marked decrease of ITIH4 fragmentation between nonunion and control patients may be attributed to two different reasons: the decreased level of ITIH4 or the decreased level of kallikrein in serum of nonunion patients. In further studies, we will determine why the enhanced or decreased ITIH4 fragmentation is selective to certain pathologies.

## Conclusions

Bones have a lifelong capacity to regenerate thanks to a perfect homeostasis between bone remodeling and repair. Bone healing in adult relies on an inflammatory process enhancing the communication between the innate immune system and local cells involved directly in the formation of bone. At the fracture site, inflammatory cells such as macrophages are mobilized to remove cell or matrix debris and to regulate tissue repair. However, a persistent or non-resolving inflammatory response may also inhibit healing and enhance the destruction of viable tissue, or sustain the production of pro-inflammatory cytokines that can impair healing. The expression of hepcidin or S100A8/S100A9 by myeloid cells and presence of complement C3, C4 and C6 could be the result of a longstanding inflammatory process. AGE products in association with a high glucose level in blood also contribute to this inflammatory process. The impaired ability to resolve the inflammatory cascade and achieve homeostasis as observed with impaired bone healing is enhanced under many conditions including smoking, obesity, diabetes, polytrauma and aging. Recent studies demonstrated that blocking macrophage activation using pharmacological agents resulted in trends towards increased callus volume and bone formation [62]. Further, mice lacking Toll-like receptor-4 (TLR-4), one of the primary receptors initiating the innate immune response, showed accelerated healing of calvarial defects [63]. Further work is required to determine if alterations in the magnitude and timing of inflammation could be targeted to improve fracture healing in at-risk patients.

## Additional files

10.1186/s12967-016-1019-1 Quality control from day 1 to day 5 using the same serum sample processed every day with the Proteominer assay.
10.1186/s12967-016-1019-1 Complementarity of spectra using different experimental conditions on the same serum sample.
10.1186/s12967-016-1019-1 Calibration process.
10.1186/s12967-016-1019-1 Most discriminant mass/charge (*m*/*z*) values obtained with the four experimental conditions used for the SELDI-TOF proteomics study.

## Abbreviations

NU: nonunion
HV: healthy volunteers
SELDI-TOF-MS: surface-enhanced laser desorption/ionization time of flight mass spectrometry
2D-DIGE: two-dimensional difference gel electrophoresis
LC–MS/MS: liquid chromatography—tandem mass spectrometry
hMSC: human marrow-derived mesenchymal stem cells
NSAID: nonsteroidal anti-inflammatory drug
CM10 arrays: cation exchange proteinchip array
IMAC-Cu: immobilized metal affinity capture bound with Cu^2+^
CHCA: α-cyano-4-hydroxycinnamic acid
PMF: peptide mass fingerprinting
ApoE: apolipoprotein E
APOA1: apolipoprotein A1
ApoA-IV: apolipoprotein A-IV
MALDI: matrix-assisted laser desorption/ionization
ITIH4: ITIH4 inter-alpha-trypsin inhibitor heavy chain
BMP-6: bone morphogenic proteins 6
TGF-β: tumor growth factor-β
PACAP: pituitary adenylate cyclase-activating peptide
PRP-48: PACAP-related peptide
PAMPS and DAMPs: pathogen- and danger-associated molecular patterns
RAGE: receptor for advanced glycation end-product
AGE: advanced glycation end-product
TLR-4: toll-like receptor 4
