# Supplementary material for: Biomarkers of inflammation and innate immunity in atrophic nonunion fracture
Source: J Transl Med. 2016 Sep 6;14(1):258. doi: 10.1186/s12967-016-1019-1 (PMC5011805; doi:10.1186/s12967-016-1019-1)

Appendix – Figure 1: Quality control from day 1 to day 5 using the same serum sample processed every day with the Proteominer assay

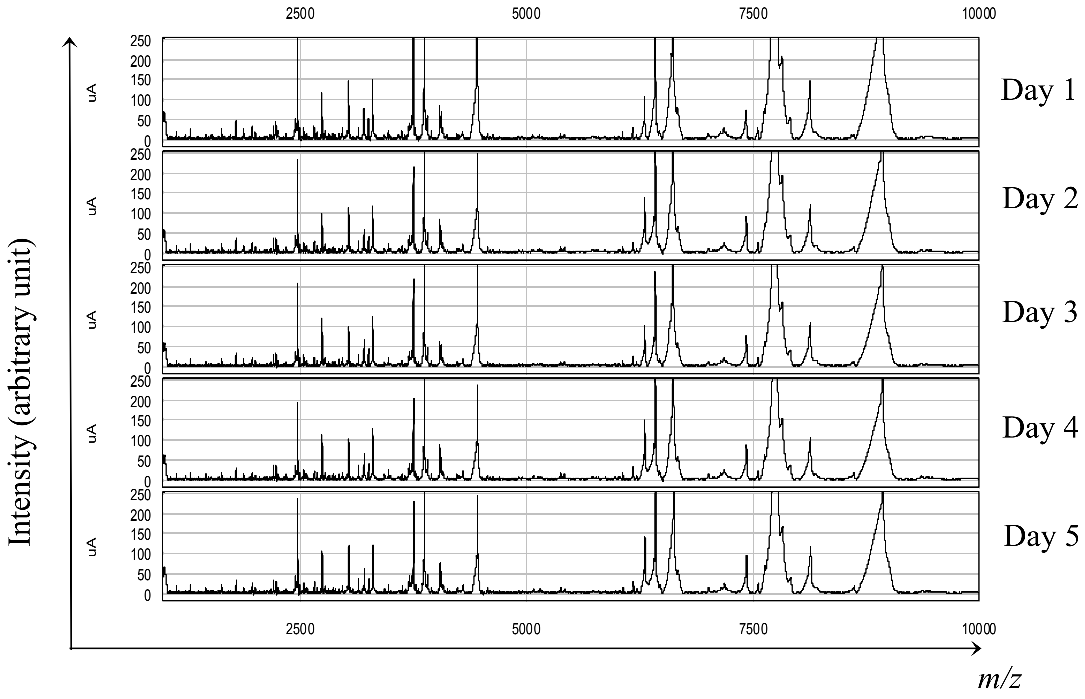

Supplement: Supplementary file 1 — 10.1186/s12967-016-1019-1 Quality control from day 1 to day 5 using the same serum sample processed every day with the Proteominer assay. [file 12967_2016_1019_MOESM1_ESM.pdf]
