# Supplementary material for: Biomarkers of inflammation and innate immunity in atrophic nonunion fracture
Source: J Transl Med. 2016 Sep 6;14(1):258. doi: 10.1186/s12967-016-1019-1 (PMC5011805; doi:10.1186/s12967-016-1019-1)

Appendix – Figure 2: Complementarity of spectra using different experimental conditions on the same serum sample. Four experimental conditions were used for the SELDI-TOF-MS proteomics study: 1) crude serum on CM10 arrays (pH9); 2) crude serum on IMAC-Cu^{2+} arrays; 3) proteominer eluate on CM10 (pH9) and 4) proteominer eluate on IMAC-Cu^{2+} arrays. All experimental conditions are illustrated with the same serum sample, and generated spectra are presented. Spectra illustrated peak intensities *vs.* mass to charge ratios (*m/z*)*:* A) from 1000 to 5000 *m/z* values; B) from 5000 to 10000 *m/z* values and C) from 10000-35000 *m/z* values.

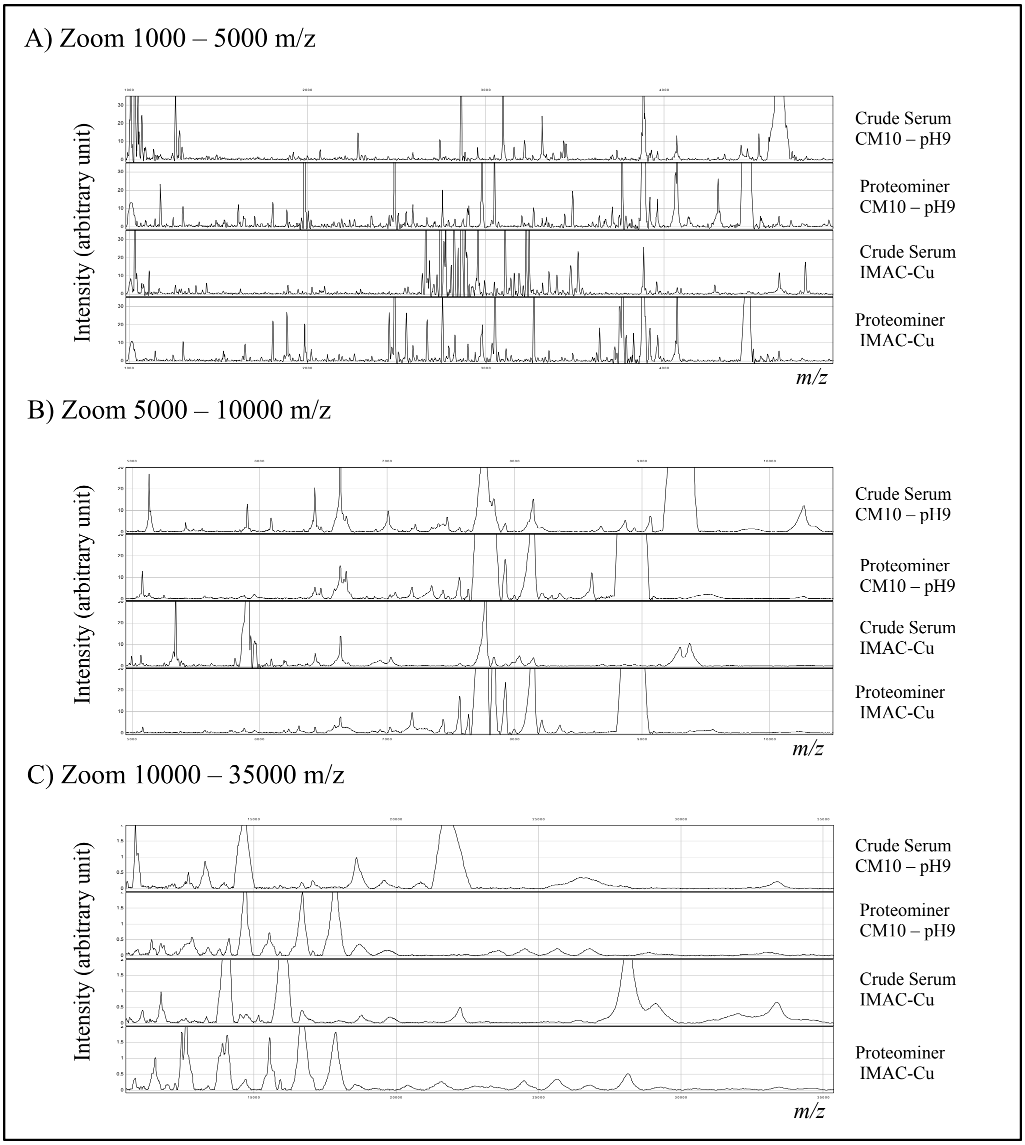

Supplement: Supplementary file 2 — 10.1186/s12967-016-1019-1 Complementarity of spectra using different experimental conditions on the same serum sample. [file 12967_2016_1019_MOESM2_ESM.pdf]
