# Supplementary material for: Biomarkers of inflammation and innate immunity in atrophic nonunion fracture
Source: J Transl Med. 2016 Sep 6;14(1):258. doi: 10.1186/s12967-016-1019-1 (PMC5011805; doi:10.1186/s12967-016-1019-1)

Appendix – Figure 3: Calibration process - Mass accuracy was calibrated externally using the All-in-1 Peptide Standard complemented with cytochrome c (MW: 12360) and myoglobin (MW 16951.5). Spectra illustrated peak intensities *vs.* mass to charge ratios (*m/z*).

Calibrants	MW	Calibrants	MW
Arg-vasopressin :	1084.25	Human insulin :	5807.65
Somatostatin :	1637.90	Hirudin, recombinant :	6963.52
Dynorphin :	2147.50	Cytochrome C (bovine):	12230.90
ACTH (1-24) :	2933.50	Myoglobin :	16951.50
Bovine Insulin (β chain):	3495.94		

Calibration : 1kDa – 10kDa

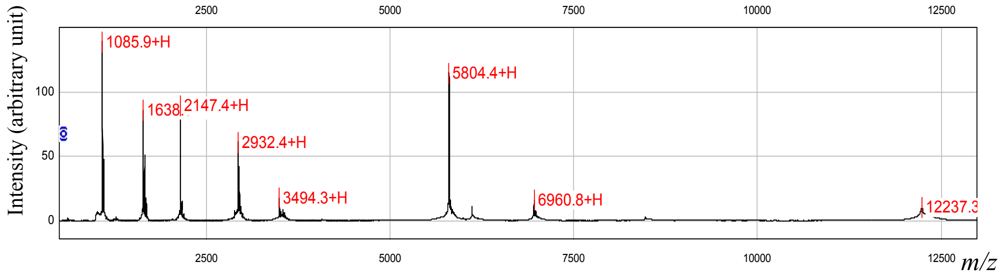

Calibration : 10kDa – 20kDa

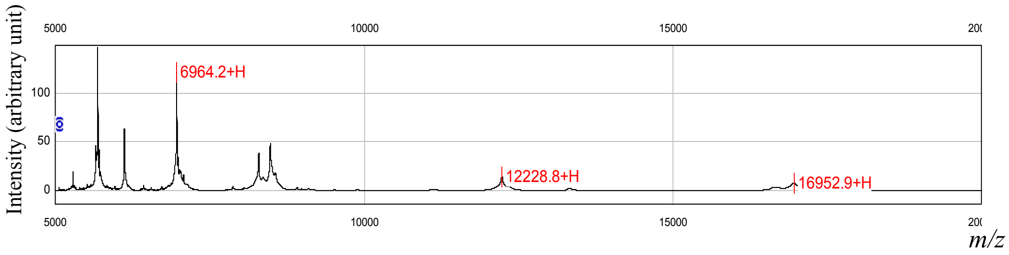

Supplement: Supplementary file 3 — 10.1186/s12967-016-1019-1 Calibration process. [file 12967_2016_1019_MOESM3_ESM.pdf]
