# Supplementary material for: Biomarkers of inflammation and innate immunity in atrophic nonunion fracture
Source: J Transl Med. 2016 Sep 6;14(1):258. doi: 10.1186/s12967-016-1019-1 (PMC5011805; doi:10.1186/s12967-016-1019-1)
Supplement: Supplementary file 4 — 10.1186/s12967-016-1019-1 Most discriminant mass/charge (m/z) values obtained with the four experimental conditions used for the SELDI-TOF proteomics study. [file 12967_2016_1019_MOESM4_ESM.pdf]

Appendix – Table 1: Most discriminant mass/charge (m/z) values obtained with the four experimental conditions used for the SELDI-TOF proteomics study: 1) crude serum on CM10 arrays (pH9); 2) crude serum on IMAC-Cu^{2+} arrays; 3) proteominer eluate on CM10 (pH9) and 4) proteominer eluate on IMAC-Cu^{2+} arrays. The relative contribution (the percentage of importance, Imp%) of each *m/z* attribute to discriminate HV *vs.* NU patients was given by the extra-trees multivariate analysis and ranked in ascending order. P-value were calculated by the non-parametric Mann-Whitney U-test, and Q-values were calculated using FDR (1000 permutations) for multiple testing. *m/z*= mass to charge; HV=healthy volunteers; NU=nonunion patients.

Crude Serum - CM10 pH9

Rank	m/z	Imp(%)	P-value	Q-value	ID
1	3179	3.79	< 0.001	0.007	ITIH4 - isoform 1 (frag 617-644) [aduct Na+]
2	7348	3.59	< 0.001	0.002	
3	2790	3.36	< 0.001	0.004	Hepcidin-25 - (frag 60-84)
4	3157	3.02	< 0.001	0.007	ITIH4 - isoform 1 (frag 617-644)
5	3539	2.77	< 0.001	0.012	
6	14636	2.24	< 0.001	0.004	
7	2321	2.14	< 0.001	0.004	
8	12112	2.11	0.006	NS	
9	4728	2.06	NS	NS	
10	5419	2.05	< 0.001	0.022	S100A8 (2H+)
11	13269	1.80	< 0.001	0.004	S100A9
12	1879	1.73	< 0.001	0.009	
13	12180	1.72	0.001	0.039	
14	29128	1.57	0.013	NS	
15	10844	1.45	< 0.001	0.008	S100A8
16	7806	1.41	NS	NS	
17	6346	1.25	< 0.001	0.013	S100A9*
18	3708	1.22	0.002	0.017	
19	3919	1.20	NS	NS	
20	4643	1.17	NS	NS	
21	4894	1.15	0.003	0.020	
22	2210	1.07	< 0.001	0.032	
23	1980	1.06	0.001	0.048	

Crude Serum - IMAC-Cu

Rank	m/z	Imp(%)	P-value	Q-value	ID
1	7994	6.03	< 0.001	< 0.001	
2	8033	5.21	< 0.001	< 0.001	Hemoglobin subunit beta + glucose (2H+)
3	7931	3.68	< 0.001	0.020	Hemoglobin subunit beta (2H+)
4	3683	3.40	< 0.001	0.014	
5	15866	2.89	0.021	NS	Hemoglobin subunit beta
6	4299	2.71	< 0.001	0.015	ITIH4 - isoform 2 (frag 617-657) [Met ox]
7	3974	2.67	< 0.001	0.020	ITIH4 - isoform 1 (frag 650-687)
8	16022	2.54	< 0.001	0.015	Hemoglobin subunit beta + glucose
9	13333	2.02	NS	NS	
10	4282	1.96	< 0.001	0.015	ITIH4 - isoform 2 (Frag 617-657)
11	25305	1.71	0.042	NS	
12	3185	1.29	NS	NS	
13	3956	1.26	< 0.001	0.012	
14	28106	1.25	0.01	0.029	ApoA1
15	8149	1.24	0.003	0.020	
16	2232	1.09	0.001	0.027	
17	2755	1.08	0.024	NS	
18	6436	1.05	0.015	0.037	
19	9664	1.00	0.029	0.077	
20	3540	0.89	< 0.001	0.015	

Proteominer - CM10 pH9

Rank	m/z	Imp(%)	P-value	Q-value	ID
1	2792	10.86	< 0.001	0.004	Hepcidin-25 - (frag 60-84)
2	8142	2.85	0.012	NS	
3	12980	2.70	0.003	0.043	
4	2540	2.40	NS	NS	
5	4798	2.31	< 0.001	0.021	PACAP-related peptide (PRP-48)
6	5956	2.17	NS	NS	
7	17052	1.76	0.001	0.027	
8	8937	1.69	NS	NS	
9	8132	1.67	0.001	0.022	C3a (frag 672-739)
10	2366	1.60	NS	NS	
11	7476	1.56	NS	NS	
12	4071	1.42	NS	NS	
13	21657	1.38	0.001	0.045	
14	8271	1.28	< 0.001	0.021	
15	4777	1.28	0.1	NS	
16	6631	1.24	0.04	NS	
17	8442	1.11	0.03	NS	
18	3486	1.11	0.005	NS	
19	7349	1.04	0.03	NS	
20	1484	1.00	0.004	NS	

Proteominer - IMAC-Cu

Rank	m/z	Imp(%)	P-value	Q-value	ID
1	10073	10.89	< 0.001	0.007	
2	5036	7.42	< 0.001	0.008	
3	2792	5.38	< 0.001	0.005	Hepcidin-25 - (frag 60-84)
4	2191	4.54	< 0.001	0.007	Hepcidin-20 - frag 65-84)
5	9314	3.09	< 0.001	NS	
6	2939	2.25	NS	NS	
7	9551	1.70	0.037	NS	
8	7479	1.43	NS	NS	
9	2569	1.40	0.03	NS	
10	1866	1.32	NS	NS	
11	4102	1.30	NS	NS	
12	28099	1.25	0.003	0.050	ApoA1
13	4924	1.17	NS	NS	
14	3289	1.02	< 0.001	0.046	
